# Primary Bilateral High-Grade Vesicoureteral Reflux in Children: Management Perspective

**DOI:** 10.7759/cureus.12266

**Published:** 2020-12-25

**Authors:** Wadha Al Qahtani, Osama Sarhan, Abdulhakim Al Otay, Ahmed El Helaly, Fouad Al Kawai

**Affiliations:** 1 Urology, King Fahad Specialist Hospital, Dammam, SAU; 2 Urology, Mansoura Urology and Nephrology Center, Mansoura, EGY; 3 Urology, Prince Sultan Military Medical City, Riyadh, SAU

**Keywords:** vesicoureteral reflux, bilateral, surgery, endoscopic injection, outcome

## Abstract

Objective

Vesicoureteral reflux (VUR) is a common finding in the pediatric population with the risk of repeated infections and renal damage. There is little is known about the natural history of primary bilateral high-grade reflux. Herein we present our experience in the management of primary high-grade bilateral VUR and the long-term outcome of renal function in this specific group of patients.

Materials and methods

We retrospectively evaluated all patients with congenital bilateral VUR between 2006 and 2014. Records were reviewed for patient age at diagnosis, antenatal history, clinical presentation, the grade of VUR on voiding cystourethrogram (VCUG), presence of scars on dimercaptosuccinic acid (DMSA) scan, indications for surgical intervention, and surgical approaches. Clinical and radiological outcomes of this subgroup of patients were assessed.

Results

A total of 67 patients with bilateral VUR were identified, of whom 31 (20 boys and 11 girls) had primary high-grade (grade IV and V) bilateral VUR. The mean age at diagnosis was seven months. DMSA scans showed renal scars in 19 patients (61%) and eight of them were bilateral. Surgical intervention was necessary for 81% of patients with a success rate of 58% after endoscopic correction and 100% after reimplantation. Chronic kidney disease (CKD) developed in 13 patients (42%) after a mean follow-up of eight years.

Conclusions

Primary bilateral high-grade VUR carries a high rate of surgical intervention. The endoscopic correction has an acceptable success rate and efficient long-term outcome. Nevertheless, a significant proportion of patients progresses to CKD even after VUR management.

## Introduction

Vesicoureteral reflux (VUR) is a common congenital anomaly characterized by either a unilateral or bilateral reflux of urine from the bladder into the upper urinary tract, with the danger of repeated urinary tract infections (UTIs) and renal damage [1,2]. It has been long recognized as a major pediatric health problem [2,3]. VUR is classified into primary or secondary according to the underlying etiology and unilateral or bilateral according to the affected side [4]. The actual incidence of bilateral VUR is not clear, however, it ranges from 30% to 58% of all children with VUR in the current literature [2,5-9].

 The clinical presentation of patients with VUR can vary significantly as some patients may present with recurrent febrile UTIs, while others can be asymptomatic. In the era of the radiological technology revolution, antenatal ultrasound became an efficient hydronephrosis screening tool. The prevalence of VUR among patients with antenatal hydronephrosis has been estimated to be 10-20%, while in normal children, the prevalence was 0.4-1.8% [10,11].

 Management options of VUR include conservative medical treatment, endoscopic correction, and anti-reflux surgery. There is no consensus on the optimal management of VUR, VUR treatment options, or the most effective timing of treatment, and because of the complexity of VUR cases and their variable presentations, the clinical management should be individualized [1,12]. Conservative treatment refers to active surveillance and antibiotic prophylaxis. Patient compliance plays a crucial role in this therapeutic option and, the patient's parent or guardian needs to understand that compliance is the cornerstone throughout the therapeutic journey of their child. On the other hand, patients on prophylactic antibiotics might experience some side effects 8-10% of the time, which can dramatically affect their compliance [7].

Surgical treatment can be classified into two main groups. The first group includes endoscopic surgeries, which refers to the endoscopic sub ureteric injection of bulking agent to create support to the intravesical ureters without obstructing the urine's antegrade flow, at the same time allowing maturation and elongation of the ureter's intramural tunnel [13-15]. The second group includes ureteral reimplantation via open, laparoscopic, and robotic approaches [15,16].

There is little is known about the natural history of primary bilateral high-grade VUR in the literature. All studies included both unilateral and bilateral cases with either primary or secondary etiology. In this study, we shed light on the patients with primary bilateral high-grade VUR as this specific group represents a high-risk group of VUR patients that can be exceptionally challenging in their management with not well-known prognosis or outcome.

## Materials and methods

We retrospectively assessed all patients with bilateral VUR below the age of 15 years in two institutions between 2006 and 2014. A total of 67 patients diagnosed with congenital bilateral VUR were identified in our medical records. Only patients with bilateral primary high-grade VUR were included in the study. Patients with bilateral primary low-grade VUR, age above 15 years, patients who lost follow up and patients with secondary VUR including posterior urethral valves, ectopic ureters, ureterocele, megaureter, or neurogenic bladder all were excluded from the study. The study was approved by the Institutional Review Board (IRB) of the two institutions.

Medical records were reviewed in regards to the age at diagnosis, antenatal history, sex, laterality, prophylactic antibiotics, breakthrough UTIs, the grade of VUR at diagnosis and at follow-up, clinical presentation, presence of scarring on DMSA, type of chosen treatment modality, clinical and radiological outcome during follow-up. The voiding cystourethrogram (VCUG) was used as an imaging diagnostic tool for all patients. The International Reflux Study in Children grading system was used to grade the reflux based on VCUG findings and high-grade VUR were grades IV and V. A baseline DMSA scans were carried out in all patients to assess renal function and to detect renal scarring.

 Patients were followed up every three to six months in the first two years and every six months later with clinical examination, blood pressure measurement, renal ultrasound, serum creatinine in addition to urine analysis and culture. A VCUG study was conducted every 12 to 24 months or earlier than that if clinically indicated. The VCUG was withdrawn from follow-up in patients with resolved VUR bilaterally or toilet trained patients who became stable with no documented UTIs (based on both clinical symptoms and urine culture). Additional DMSA scans were required in patients who developed repeated febrile UTIs or patients with renal function deterioration.

Clinical and radiological outcomes were assessed. The resolution of VUR in our study was considered when the VCUG study became normal and no more reflux bilaterally on at least one study. The downgrading of VUR was defined as grade reduction from high-grade VUR bilaterally to low-grade bilaterally (grade I-II). Renal function was assessed by determination of serum creatinine levels and the estimation of the glomerular filtration rate (eGFR) by the Schwartz formula [17]. The stage of CKD was determined according to recommendations from the National Kidney Foundation [18]. Chronic kidney disease (CKD) was defined as an eGFR <90 mL/min/1.73 m2.

Conservative management was initiated in all patients at initial presentation and it comprises active surveillance with prophylactic antibiotics and treatment of associated bowel dysfunction. For neonates born at our institution with antenatal HN, antibiotic prophylaxis was initiated using amoxicillin at doses of 100 mg/day. Starting at three months of age, the drug used for chemoprophylaxis was nitrofurantoin (1-2 mg/kg/day) or trimethoprim (2-4 mg/kg/day).

Surgery was done for patients with repeated breakthrough infections, reflux nephropathy, worsening in renal function, or presence of new scars on DMSA. A detailed explanation of potential surgical risk and benefit for both open re-implantation and endoscopic surgery was conveyed well to the patient’s parents or guardians. We have used dextranomer gel/hyaluronic acid (Dx/HA), (Deflux®; Q-Med Scandinavia, Uppsala, Sweden) as a bulking agent first-line treatment in 24 patients while open intravesical cross trigonal uretero-vesical reimplantation (Cohen's technique) primarily in one patient and secondarily in three. Treatment success was assessed postoperatively in all patients using the VCUG study carried out six to nine months postoperatively.

Data obtained from the medical records were exported into Excel for data manipulation and cleaning, then the final data loaded into Statistical Package for the Social Sciences (SPSS) software (IBM SPSS Statistics, Chicago, IL, USA), and descriptive statistical analysis was done. For quantitative data, the mean and standard deviation (SD) were calculated. Categorical variables were expressed as numbers and percentages, and for comparison, a Chi-square test was executed. P-value < 0.05 was considered to be statistically significant.

## Results

Out of 67 patients with bilateral VUR, 31 patients were found to have primary bilateral high-grade VUR (grade IV and V). The mean age at diagnosis was seven months. Male patients accounted for 64% of the cases (20 out of 31) and female patients for 36% (11 out of 31). All male patients underwent routine neonatal circumcision and all of them were on prophylactic antibiotics. The mean initial serum creatinine was 46 ± 17 µmol/L and the mean initial eGFR was 110 ± 26 mL/min. The mean follow-up period was 8 ± 3.5 years. 

The clinical presentations were antenatal hydronephrosis with varying degrees in 19 patients, UTIs in 8, CKD in three, and neonatal sepsis in one patient. Out of 31 patients, 26 patients were observed to have bilateral hydronephrosis with variable degrees on renal ultrasound. DMSA scans showed renal parenchymal scars in 19 patients (61%), and eight of them were bilateral scars. Decreased cortical uptake (split function less than 40%) on DMSA was found in 45% of the cases (14/31), and 55% had a split renal function of more than 40% (17/31). Patients’ characteristics are shown in Table 1.

**Table 1:** The demographic data of our study patients DMSA: dimercaptosuccinic acid; CKD: chronic kidney disease; HN: hydronephrosis; SFU: Society for Fetal Urology

Variable		Number (31)	Percentage (%)
Mode of Presentation	Antenatal Screening	19	61
	Urinary Tract Infection	8	26
	CKD	3	10
	Neonatal Sepsis	1	3
Gender	Male	20	64.5
	Female	11	35.5
Renal Ultrasound	No HN	3	10
	Low Grade HN (SFU 1,2)	16	52
	High Grade HN (SFU 3,4)	12	38
Renal Scarring on DMSA	No Scars	12	39
	Scars	19	61
Split Function on DMSA	< 40%	14	45
	> 40%	17	55
Management Approach	Conservative	6	20
	Endoscopic Injection	24	77
	Open Surgery	1	3

**Figure 1:**
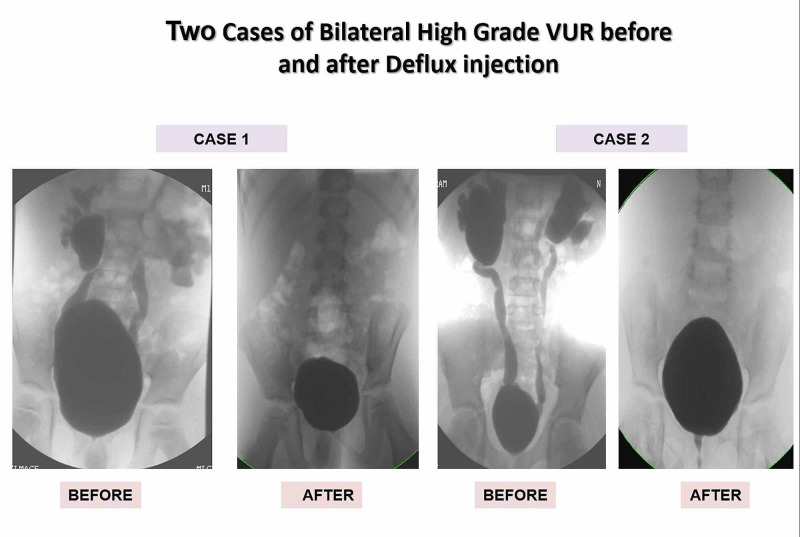
Deflux injection cases VUR: vesicoureteral reflux

Surgical intervention has been carried out in 81% of patients (25/31); 24 of them underwent endoscopic injection of bulking agent dextranomer gel/hyaluronic acid (Dx/HA) 2-5 mL in amount and one patient underwent immediate open surgery. The mean age at surgical intervention was 45 months (range, 9-112). The success rate in the endoscopy group was 58% and downgraded to low-grade VUR (grade I and II) in 17% of the cases (Figure 1).

**Figure 2:**
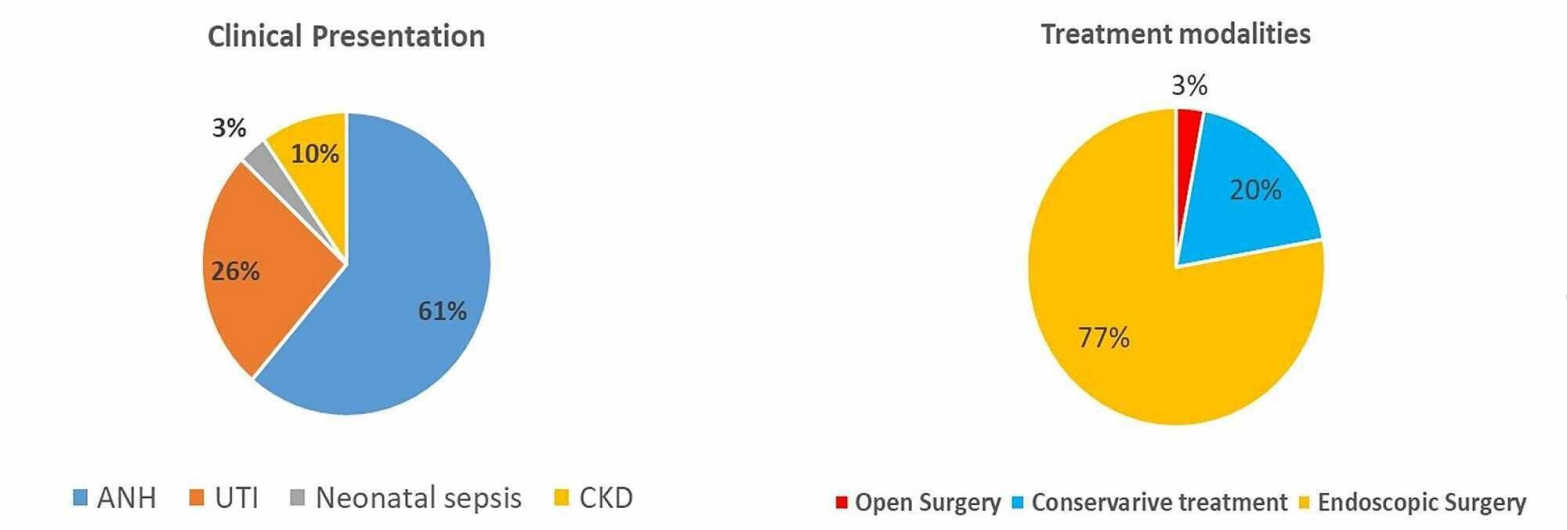
Clinical presentation and treatment modalities ANH: antenatal hydronephrosis; UTI: urinary tract infection; CKD: chronic kidney disease

One patient required redo-endoscopic injection due to persistent bilateral VUR with repeated UTIs, which resulted in complete VUR resolution bilaterally. Subsequent open reimplantation was necessary for three patients after failed endoscopic injection due to persistence of symptoms or deterioration of renal function. The success rate after open surgery was 100% (Figure 2).

A significant association was not observed between patients’ gender, their mode of diagnosis, or their need for surgical intervention. Patients with renal parenchymal scarring were referred to surgical treatment in nearly 90% of the cases while 66% of patients with normal DMSA scan required surgical intervention, however, the difference was not statistically significant (p=0.11).

Complete resolution of VUR was evident in 19 of our patients (61%) at the last follow-up. Among the patients who were on conservative treatment, the resolution rate was 67% while in patients who underwent surgical correction the resolution rate was 58%. Additional down-grading of VUR was attained in 4 patients after one or two endoscopic injections. There was no significant correlation between the mode of presentation, patient gender, DMSA findings, and rate of VUR resolution.

The mean serum creatinine in patients at the last follow-up was 73 ± 40 µmol/L while the mean eGFR was 92 ± 36 mL/min. The incidence of CKD, stage 2 or more, in our patients after a mean follow-up of eight years was 42% (13 out of 31), six of them had no VUR at the last follow-up. The remaining 18 patients maintained normal renal function at the last follow-up. When we compared the incidence of CKD in relation to patient gender, mode of presentation, and renal scarring and SRF on DMSA, comparable rates of CKD were observed and none of these variables had any significant impact on the occurrence of CKD in this group of patients.

In fact, patients who underwent surgical intervention were observed to have a relatively higher incidence of CKD and lower VUR resolution rate than those with conservative treatment but the difference between the two groups did not reach a statistical significance. Patients’ variables in relation to surgical intervention prospect, renal function outcome, and VUR resolution are shown in Table 2. Three patients (10%) developed high blood pressure during follow-up and were managed by appropriate antihypertensive medications.

**Table 2:** Patients’ variables in relation to surgical intervention prospect, renal function outcome, and VUR resolution VUR: vesicoureteral reflux; DMSA: dimercaptosuccinic acid; CKD: chronic kidney disease

Variable		Surgical Intervention Number (%)	P-Value	Incidence of CKD Number (%)	P-Value	VUR Resolution Number (%)	P-Value
Mode of Presentation	Antenatal Dx	15/19 (79)	0.763	9/19 (47)	0.430	12/19 (63)	0.788
	Postnatal Dx	10/12 (83)		48/12 (33)		7/12 (58)	
Gender	Male	17/20 (85)	0.690	8/20 (40)	0.530	13/20 (65)	0.919
	Female	8/11 (73)		5/11 (45.5)		6/11 (54.5)	
Renal Scarring on DMSA	No Scars	8/12 (66.5)	0.117	4/12 (33)	0.440	8/12 (66.5)	0.625
	Scars	17/19 (89.5)		9/19 (31)		11/19 (58)	
Split Function on DMSA	< 40%	11/14 (78.5)	0.791	6/14 (43)	0.925	9/14 (64)	0.756
	> 40%	14/17 (82)		7/17 (41)		10/17 (59)	
Management Approach	Conservative	0/6 (0)		2/6 (33)	0.634	4/6 (66.5)	0.762
	Surgical	25/25 (100)		11/25 (44)		15/25 (60)	

## Discussion

The management of VUR is controversial and should be individualized according to each patient's risk stratification. The goals of management are mainly symptomatic in terms of prevention of recurrent febrile UTIs, prevention of renal damage, and reduction of treatment morbidity [1,19,20]. Different management options for VUR include conservative treatment, endoscopic injection of several bulking agents, and ureteral implantation via different surgical approaches [1,13,16]. Management of VUR has become more conservative over time as treatments have not been reliably proven to prevent renal scarring. Bilateral and high-grade VUR were considered as high-risk factors that necessitate a more aggressive approach [12,19]. There is a lack in the literature for studies concerning primary bilateral VUR management and prognosis. Herein we present our experience in the management of primary high-grade bilateral VUR and to assess the long-term outcome of renal function in this specific group of patients.

VUR, especially with coexistent UTIs, has been associated with increased risk of renal scarring, proteinuria, hypertension, and CKD and the higher the grade the higher will be this risk. Conservative treatment with anti-microbial prophylaxis (CAP) seeks to maintain sterile urine rendering VUR harmless as no bacteria will reach and invade the kidneys. Management of VUR has been shifted from initial surgical correction into an initial period of observation with CAP to allow for spontaneous resolution [1,19,20]. Recent studies showed the significance of CAP in reducing the incidence of febrile UTIs in high-grade VUR [19-22]. In addition, several studies did not show any difference in the overall incidence of UTIs and renal scarring in patients with VUR under medical versus surgical management. We are not able yet to determine which patients will benefit from aggressive treatment of their VUR.

In this subgroup of patients with high-grade bilateral VUR, all patients were initially managed conservatively with watchful waiting and CAP. We reported a high rate of spontaneous resolution (66%) and this exceptionally high rate may be due to a small number of cases who underwent conservative treatment. High-grade VUR detected early in life has a high probability for spontaneous resolution over time [21,23]. A high incidence of spontaneous resolution up to 50% was reported in some studies like our findings [2,8,9]. In contrast, other studies showed a decreased rate of resolution in higher grades of VUR between 5-23% [5,6]. Garcia Roig and colleagues reported that high grade (IV or V) reflux was not associated with a resolution at any point [24].

Traditionally, unilateral reflux was thought to have a higher resolution rate and bilaterality was considered as a high-risk factor when we stratify risks in VUR patients [12]. In fact, we could not find any differences in the rates of spontaneous VUR resolution between unilateral and bilateral VUR in many studies including a large number of cases [5,6,8,21,24]. Meanwhile, Estrada et al. (2009) documented that unilateral VUR resolved earlier than bilateral VUR in 2462 patients (57% of them bilateral) with a 37% resolution rate after short-term follow-up [9].

Likewise, patients with high-grade VUR detected early in life are more likely to undergo surgical correction [24]. Anti-reflux surgery aims to block the abnormal ascent of urine and bladder infection to the upper tract by reconfiguring the anatomy of the uretero-vesical junction [16,19,25]. Surgical intervention was indicated in 81.5% of our patients due to repeated breakthrough febrile UTIs, deterioration of renal function, or persistence of high-grade VUR. This high rate for intervention was expected as this subgroup of patients with high-grade bilateral VUR showed a high probability of repeated UTIs and the development of renal scarring. In the study of Alvarez et al. (2018) [26], the percentage of patients with high-grade VUR who underwent surgery was higher (81.25%) than those with low-intermediate grade VUR (18.75%). Teixeira et al. stated that patients with renal scarring were predominantly referred to surgical correction and most of our patients had renal scarring at initial DMSA scans [7].

Surgical intervention indications are usually according to the previously reported guidelines [1,19,20]. The rate of surgical intervention in high-grade VUR patients was reported between 36% and 81% [3,7-9,21-26]. Surgical correction was necessary for breakthrough UTIs or persistent VUR in 50-89% of cases according to the VUR index score [24]. Teixeira et al. (2014) described that 68% of patients underwent surgical correction of which 73% of cases of bilateral VUR underwent surgical intervention [7]. Furthermore, Alvarez et al. stated that patients with high-grade VUR required surgery in a significantly greater proportion due to recurrent febrile UTIs [26].

Since first reported by Stenberg and Lackgren in 1995, Dx/HA copolymer has been the most commonly used product for VUR injection treatment in children and the only treatment approved by the Food and Drug Administration [27]. The endoscopic injection was effective at eliminating VUR in children, even in patients with high-grade VUR, as well as in patients with VUR and additional malformations. Success rates of Dx/HA copolymer injection in high-grade VUR were reported between 50% and 60% parallel to our results [13,15,19,28].

The rate of open surgical correction is decreasing nowadays with the development of laparoscopic and robotic approaches [16,19]. Open uretero-vesical reimplantation had a success rate of over 90-95%. We have only one patient who underwent initial open surgical correction and 3 patients after initial endoscopic injection with a success rate of 100%.

Congenital anomalies of the urinary tract are an important cause of CKD in children and reflux nephropathy accounts for 7-17% of ESRD worldwide and the impact of surgical intervention in the protection of renal function is still vague [29]. Several studies have demonstrated the lack of benefits in renal function in those patients who undergo surgery at diagnosis, in terms of appearance and progression of renal scars, hypertension, or renal insufficiency [22,26].

That is always the concern what renal changes occur because of high-grade VUR versus that which is a result of the overall embryologic pathology. Our findings demonstrated that surgical correction of VUR did not directly impact the development of CKD points toward that renal pathology which is already presently leading to renal decline versus the development of pathology as a result of VUR.

Decreased renal function is more frequent in patients with high-grade VUR [26]. Sjostrom (2010) reported 39% abnormal GFR in 115 patients with VUR [21]. In our study, CKD developed in 42% of our patients. Impaired renal function was significantly related to renal scarring, especially in bilateral VUR.

High-grade VUR has been associated with a high risk of developing renal scars and confer a great incidence of abnormal DMSA with age [21,30]. Some congenital defects may represent congenitally rather than acquired renal damage particularly in high-grade VUR which makes the distinction between the two types impossible [30]. The incidence of renal scarring in our patients was 61%, of whom eight were bilateral. Comparable incidence was showed by Sjostrom et al who observed abnormal DMSA scans in 63% of grade IV and 67% of grade V reflux [21,23]. In another study by Snodgrass and colleagues on 565 patients with VUR, 50% of cortical defects on DMSA were found in grade IV and V patients [30].

Limitations of our study included its retrospective nature with inherent selection bias and small sample size that led to a non-significant statistical difference from statistical tests applied in between the study group. Indications for surgical intervention were not unified in all cases and the approach depended on surgeon preference and family acceptance.

## Conclusions

This study showed that patients with primary bilateral high-grade VUR carry a high rate of surgical intervention. Endoscopic surgeries either by single or multiple injections can be a proper treatment option in those patients with an acceptable success rate and an efficient long-term outcome. A significant percentage of patients developed CKD even after VUR resolution and, noteworthy, that most of them had renal parenchymal scars on the DMSA scan. These associated DMSA findings could be related to developmental hypoplasia and rather than UTIs. There were no significant variables in this subgroup of patients that could predict the need for surgical intervention, resolution rate, or ultimate renal function.
